# An Editor’s Special

**Published:** 1884-11

**Authors:** 


### Editorial.

### AN EDITOR'S SPECIAL.

Perhaps we are not as thankful as we should be for the grandfatherly care that some of our good journalistic friends manifest a desire to exercise over us, but as we do not feel that the responsibility for the carefully cultivated eccentricities of language, or the persistent thrusting forward of the great "I" by the respected editor of the *Ohio Dental Journal* rests upon our shoulders, we cannot see why he should be so troubled about our shortcomings. It is true, perhaps, that the editor of this Journal "has scarcely begun his career" (though we have been "careering" for more years than we like to reckon up), but we will try and find some consolation in the reflection that a possible immaturity is a more hopeful condition than a positive dotage. There is certainly abundant opportunity, and we trust there is in us an honest desire, for improvement. To this end we gladly accept reproof and correction from any one who can demonstrate his title as Master. But the chemist who teaches that dental caries is due to mineral acids, the physiologist who insists that he has caused a dog to breathe pure nitrous oxide gas exclusively for an hour without deleterious effects, the histologist who warns dentists to exercise caution in the extraction of deciduous teeth because there is danger of pulling out the germ of the permanent one that may be adherent to it, does not seem to one just entering upon his "career" quite a safe guide to follow. Age grants privileges but not license, and though it may bring experience it does not always endow with wisdom. If we are to conform ourselves to some type we wish a perfect one, and —considering the circumstances our very much respected friend will pardon us if we are becoming a little restive under his repeated corrections and continued discipline.

